# Developing an Australian-first recovery model for parents in Victorian mental health and family services: a study protocol for a randomised controlled trial

**DOI:** 10.1186/s12888-017-1357-4

**Published:** 2017-05-26

**Authors:** Darryl Maybery, Melinda Goodyear, Andrea Reupert, Jade Sheen, Warren Cann, Kim Dalziel, Phillip Tchernagovski, Brendan O’Hanlon, Henry von Doussa

**Affiliations:** 1 Department of Rural Health, Monash University, Rural Mental Health, PO Box 973, Moe, VIC 3825 Australia; 2 School of Rural Health, Monash University, Clayton, VIC 3800 Australia; 3 Krongold Faculty of Education, Monash University, 57 Scenic Boulevard, Clayton, 3168 Australia; 4 Deakin University, Burwood, Australia; 5 Parenting Research Centre, Level 5, 232 Victoria Pde, East Melbourne, 3002 Australia; 6 Melbourne School of Global and Population Health, The University of Melbourne, Melbourne, VIC Australia; 7 Department of Rural Health, Monash University, PO Box 973, Moe, VIC 3825 Australia; 8 The Bouverie Centre, La Trobe University, 8 Gardiner Street, Brunswick, VIC 3056 Australia; 9 Faculty of Medicine, Nursing and Health Sciences, Monash University, Clayton, VIC Australia

**Keywords:** Mental illness, Parent, Family, Randomised controlled trial, Protocol

## Abstract

**Background:**

A considerable number of people with a mental illness are parents caring for dependent children. For those with a mental illness, parenting can provide a sense of competence, belonging, identity and hope and hence is well aligned to the concept of personal recovery. However, little research has focused on the recovery journey of those who are parents and have a mental illness. This randomised controlled trial aims to (i) evaluate the effectiveness of an intervention model of recovery for parents (Let’s Talk about Children) in three different mental health service sectors and (ii) examine the economic value of a larger roll out (longer term) of the parent recovery model.

**Methods:**

A two arm parallel randomised controlled trial will be used with participants, who are being treated for their mental illness in adult mental health, non-government community mental health or family welfare services. The study will involve 192 parents, who are considered by their treating practitioner to be sufficiently well to provide informed consent and participate in an intervention (Let’s Talk about Children) or control group (treatment as usual). Participant randomisation will occur at the level of the treating practitioner and will be based on whether the randomised practitioner is trained in the intervention. Outcomes are compared at pre, post intervention and six-month follow-up. Recovery, parenting and family functioning, and quality of life questionnaires will be used to measure parent wellbeing and the economic benefits of the intervention.

**Discussion:**

This is the first randomised controlled trial to investigate the efficacy of a parenting intervention on recovery outcomes and the first to provide an economic evaluation of an intervention for parents with a mental illness. An implementation model is required to embed the intervention in different sectors.

**Trial registration:**

The trial was retrospectively registered: ACTRN12616000460404 on the 8/4/2016.

## Background

A significant proportion of individuals with a mental illness are parents who are caring for dependent children. Nicholson [1] found that 38% of women with serious mental illness were mothers, while another study [2] estimated that 21 and 23% of all families have at least one parent who has (or had) a mental illness. Parenting is a valued social role and for those with a mental illness provides meaning, purpose and identity [1] all core components of a personal recovery approach. Thus, a recovery intervention that encapsulates a service user’s parenting role and responsibilities holds much promise.

While the medical model of recovery emphasises the remission of mental health symptoms, the personal recovery model has been defined as a ‘change in outlook that is related to leading a meaningful, purposeful life, with or without ongoing episodes of illness’ [3].^(p268)^ Personal recovery is a complex concept that encompasses multiple life domains such as community engagement and employment [4], however little attention has been paid in the literature to the role of parenting within personal recovery frameworks [5]. Nicholson [1] found that children give parents the strength and will to ‘keep going’ thereby promoting hope (a key element of recovery). ‘Being a parent’ and effectively assuming the parenting role, provides parents with meaning and purpose (another element of recovery [1]). Parenting may also contribute positively to individuals’ lives by providing opportunities for meaningful interactions and activities [5]. Thus, identifying and supporting an individual’s parenting role can provide hope, a sense of agency, self-determination and meaning, all aligned with a recovery approach.

While there are some, albeit limited, evidence based interventions designed for families where a parent with a mental illness, approaches rarely consider how recovery and parenting might be aligned. Instead, most appear to be formalised interventions that promote psycho-education and family communication and, on the whole, are predominately designed for mothers with affective disorders [6]. There is, however, emerging evidence as to the effectiveness of interventions for parents with a mental illness and/or their children; Siegenthaler, Munder and Egger [7] employed a systematic review and meta-analysis of the effectiveness of 13 interventions designed to prevent mental illness in the children of parents with a mental illness. Across the 13 trials including 1490 children of parents with mental illness, interventions decreased the risk of developing mental illness for such children by up to 40%, predominantly through parent or family mediated programs [7].

One of the studies reviewed by Siegenthaler was the Let’s Talk about Children (Let’s Talk) approach. Let’s Talk is a two to three session intervention designed for parents with the mental illness [8]. In the intervention, parents are empowered to develop their own strategies to promote child and family strengths and discuss ways to talk to their children about their mental illness. In this process, practitioners engage with their parent-client in a discussion around the children that focusses on their strengths and any concerns the parent may have about their child. While other family members might be involved in these sessions, the focus is typically on empowering the parent with a mental illness in his or her parenting role. This clinical stance seeks to empower parents, so they may acquire the confidence, understanding and skills to promote change in their family. The program has been previously trialled in over two thirds of Finland’s health regions [9], with one RCT study reporting an increase in parents’ understanding of the impact of mental illness and on reductions in children and parents’ guilt, shame and perceived prejudice [8, 9]. Notably, training practitioners in the intervention led to significant practice changes, with a 16% increase in onward referrals for children to other services following the intervention [8].

Given the focus of Let’s Talk on parent empowerment and family strengths, the intervention is well aligned to personal recovery, relevant to parents with various disorders and levels of disability and applicable to different workforce sectors. How Let’s Talk impacts on recovery has not been investigated. Additionally, as highlighted by Bee and colleagues [10] no economic evaluations, cost or resource-use studies have been conducted in this area, an often critical consideration for governments when allocating funding and resources.

This study will examine the impact of an Australian-first parent recovery approach that incorporates Let’s Talk across three service sectors. The three service sectors include adult mental health, community mental health and family services in Victoria, Australia. Adult mental health services are a specialist clinical public service for people with a severe mental illness, including significant levels of disturbance and psycho-social disability; the service includes acute inpatient and continuing care facilities. The community mental health sector, sometimes known as psychiatric rehabilitation, includes non-government mental health community support services, designed for those with a chronic mental illness. Community practitioners work collaboratively with their clients on individual support plans, that are recovery focused. The family services sector offers support to vulnerable families with children at-risk, where risk factors include parental mental illness, family conflict or breakdown and problems related to child development, school attendance and social or economic disadvantage. Clients across all three sectors present with a wide range of mental illnesses including psychotic disorders, bipolar disorder, as well as affective disorders. However, parents in the family service sector, compared to the other sectors, are generally considered to present with less severe mental health problems, but with a high comorbidity of family violence and alcohol and other drug issues. The Let’s Talk approach is considered suitable for all levels of mental illness severity (T. Solantaus 2013, personal communication) but requires stability of symptoms for parents to engage in the intervention. Those parents in an acute stage of symptom presentation are recommended not to engage in the intervention.

The research will establish an evidence base for a recovery model that effectively addresses parenting, as measured by adult recovery (e.g. quality of life, hope, social inclusion), parenting competence and self-efficacy, family functioning. It is hypothesized that:

- Significant improvements (*p* < 0.5) will be reported by parents on the eight psycho-social dimensions of recovery
- Significant improvements (*p* < 0.5) will be reported on parenting sense of competence and parenting self-efficacy
- Significant improvements (*p* < 0.5) will be reported on family functioning.

The study will also examine the economic value of a larger roll out (longer term) of the parent recovery model for investment by Victorian and Australian governments.

## Methods

### Design

This two-arm parallel randomised controlled trial will compare outcomes at post-test and 6 month follow-up (Trial register number ACTRN12616000460404) for Let’s Talk and control participants from the adult mental health, community mental health and family services. The protocol is in accord with the CONSORT-EHEALTH checklist. The study commenced in 2013 and will be completed in 2017.

### Study population

Clients who are parents in treatment at adult mental health, community mental health and family services sectors in Victoria, Australia will be the participants in this study. Mental illness is defined according to DSM-5 [11]; as a clinically significant disturbance in an individual’s cognition, emotion regulation, or behaviour that reflects a dysfunction in the psychological, biological, or developmental processes underlying mental functioning. Client eligibility includes; a mental illness diagnosed by either a GP or mental health practitioner, be between the ages of 18 and 65, have at least 20% custody of at least one child under 18 years of age, be attending services for their mental health problems, able to comprehend English and deemed (by their treating practitioner) to have the mental health capacity to participate in the research and intervention.

### Recruitment

Participating organisations will be recruited through an expression of interest process and public forums. Practitioners will be nominated by the organisation if they show an interest in family sensitive practice and/or have parent-clients with mental health issues on their current caseload. Eligible parents will be invited by their treating practitioner to be involved in a research study about recovery and families. At this point they will not be given any information about the intervention. The practitioner will give their parent-client a brief introduction to the research including a consent form. No information will be given to participants about the specific intervention as a description of the intervention (which highlights the parenting role and the needs of children) may possibly influence participants, particularly those who may not have considered or discussed their parenting role in relation to their illness. Parents interested in participating in the research will then authorise the practitioner to forward the parent’s contact details to the researchers. The researchers will then contact the parent-client (e.g. by phone, email) with comprehensive information (including written) of the research (not the Let’s Talk intervention) and will obtain consent from those interested in continuing in the research study. At this point, potential participants will be told that the research is about recovery and mental illness (not about parenting and children). All consenting parents will be sent a questionnaire pack and a reply paid envelope and will be offered phone assistance to complete questionnaires.

Following parent completion of the first questionnaire pack, the practitioner will be randomised to either the (1) wait list control group or (2) to receive training and deliver the Let’s Talk intervention. Randomisation has been designed to occur at the practitioner level to address any potential effects of attending Let’s Talk training in the control condition. The researchers will be blind to the allocation of the practitioner, with an independent administrative officer (based at a different site to the research team) allocating practitioners to groups based on the computer generated random sequence (using SPSS). Within 1 month after being allocated, Let’s Talk practitioners will be provided with training in the intervention. The control practitioners will continue treatment as usual and will not be trained in the intervention until completion of the study. Practitioners trained in the intervention will then offer Let’s Talk to their clients. Those parent-clients who decline the intervention will continue with treatment as usual with their practitioner (and continue in the research study if they wish as a separate comparison group in the analysis). Both wait-list control and Let’s Talk trained clinicians may refer any number of eligible parent-clients to the study who indicate interest in the research.

The control group will continue to receive ‘treatment as usual’ from their non-trained (in Let’s Talk) practitioner and will be informed about (and offered) Let’s Talk as they complete the final follow up questionnaire (following the training of their treating practitioner). Treatment as usual refers to the standard treatment or model of care usually provided to clients of each of the services involved in the trial. For the purposes of the trial this will be identified as treatment as usual in adult mental health clinical services, non-government mental health community support services and family support services.

### Randomisation and blinding

Participants will be randomly identified using a random number generator (SPSS) to intervention and control conditions. Permutation blocks of 36 will be used to ensure a minimum number of Let’s Talk and control group allocations.

### Data collection

Participants will complete measures prior to randomisation and 6 weeks post the completion of the Let’s Talk intervention (including an equivalent time frame for the control group) and 6 months after the post test. Participants will have the option to complete their questionnaires by hand (and post their questionnaire), verbally over the phone or in person with one of the members of the research team.

### Let’s talk practitioner training

Practitioners are trained in Let’s Talk by completing four online modules (1 h each) [12] followed by a 4 h face to face training session facilitated by experienced trainers within 4 weeks of completing the online modules.

### Wait-list control group/standard treatment

Participants in the control condition will receive treatment as usual from a practitioner who has not been trained in the Let’s Talk model (Table 1).

### Outcome measures

The primary outcome measures included the *Parental Stress Scale* [13] that measures perceived parenting stress and includes 18 items that together measure total parenting stress along with four subscales of parental rewards, parental stressors, lack of control and parental satisfaction. The *General Functioning Index* of the *McMaster Family Assessment Device* [14] measures family functioning. The General Functioning Index consists of 12 items and measures an overall score of family functioning that distinguishes between ‘effective and problematic’ family functioning. The *SF-12v2 Health Survey* [15] will be used as a measure of quality of life. The survey is a 12 item quantitative instrument that records impacts of mental and physical health on daily activities. Outcomes were measured at 6 months followup.

The secondary outcome measures were the *Parenting Self-Agency Measure* [16] that assesses the general level of confidence parents have in their ability to engage in successful parenting behaviours. The measure examines the domains of confidence, helplessness in the face of child opposition, ability to resolve parent-child conflict, and effort in parenting and persistence. The *Recovery Assessment Scale* [17] is a 22-item recovery scale commonly used in mental health services. It includes five subscales as well as a total score: Personal confidence and hope; Willingness to ask for help; Goal and success orientation; Reliance on others; and No domination by symptoms. Each of the above measures has been widely used and are well validated and exhibit strong internal consistency.

#### Economic evaluation

An economic evaluation will be conducted examining the costs of delivering the program (sourced from practitioner records) combined with costs for individuals of accessing treatment (using retrospective self-reported medication and hospital use along with patient and carer time away from usual duties). The evaluation takes a societal perspective including financial implications to the health system and individual patients and their carers. Outcomes will consist of the trial measures: impact on disease recovery rates and quality of life impacts (converting the SF-12 scores to SF-6D utility measures suitable for economic evaluation [18]. Incremental cost-effectiveness will involve the comparison between intervention and control groups of cost per additional person recovered and cost per quality adjusted life year gained using impacts from the SF-12. The cost-effectiveness model will take the form of a decision analysis modelling people between mentally ill and recovered health states, with results presented initially for 12 months follow up. Modelling to full life expectancy will be performed incorporating lifetime costs associated with mental illness sourced from the scientific literature. Extensive one-way and probabilistic sensitivity analyses will be conducted to assess the impact of uncertainty on results.

#### Fidelity adherence

Fidelity to the Let’s Talk method will be measured through multiple means. Firstly, trained practitioners will be required to attend monthly practice development sessions to report on their delivery of the intervention in practice. Secondly, practitioners will be required to complete a fidelity checklist recording each session, the duration and content of the session, as part of their standard record keeping. These checklists will be provided to the researchers at the completion of the intervention, and used as a measure of fidelity of the delivery of the intervention.

**Table 1:**
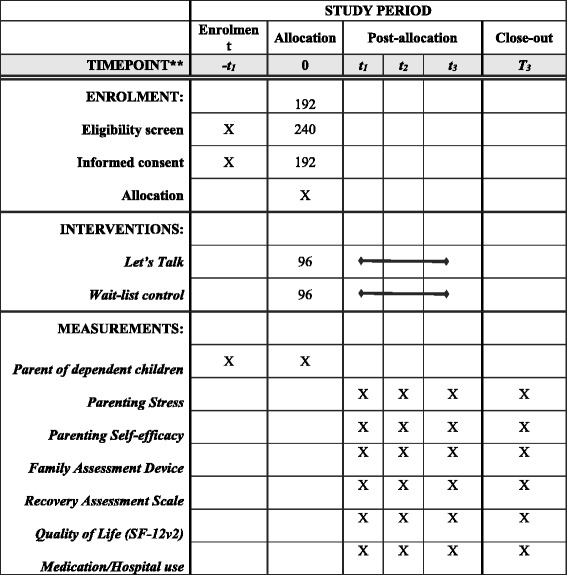
Overview of the research protocol for numbers to be enrolled, the intervention delivered, and measurement tools utilized throughout the study

t_1_ refers to the baseline measure, t_2_ is the post intervention or equivalent measure, and t_3_ is the 6 month follow-up measure post intervention

### Participant numbers

A total of 192 parents will be recruited. Participant numbers were initially determined by a power calculation indicating a minimum *n* = 54 participants with Crit F = 2.46 (using GPOWER 3.1, assuming 3 repetitions, a small effect size, an alpha of 5% and power of 95%) to be required. The larger number of participants will account for parent dropout and to allow between group comparisons across sectors (*n* = 32 per sector per group for both intervention and control groups).

## Design and statistical analysis

### Quantitative data analysis

The impact of Let’s Talk on parent-clients will be examined pre, post and follow-up using analyses of variance on each of the measures outlined above. There will be a within-subjects factor (time) and a between subjects factor (intervention/control and sector of participants – adult, community and family sectors). It is intended that analyses will be undertaken on intention to treat data however deviations from protocol adherence will also be examined including any differences between participant groups on demographic characteristics.

## Ethics and dissemination

Ethics approval for this study was obtained from Monash University Human Research Ethics Committee on January 29 2014 (approval number CF13/3300120130017) along with seven other regional health authorities. The outcomes of the trial will be disseminated at conferences and in peer reviewed journals. The general public including parents with a mental illness, their families, mental health practitioners and policy makers, will be notified of the study through public forums, government reports, policy statements, newsletters and social media.

## Limitations

It is noted that practitioners recruited to the study may have a strong interest in family focused practice and/or have previously worked with parents with mental illnesses. Thus trial outcomes may potentially be biased by a practitioner’s willingness to already engage in the concerns of parents with mental illness. However, the nature of the randomisation of practitioners to receive training should address this concern.

Parents’ diagnoses will be self-reported by the participating parent, and is not independently verified. Parents will be asked who provided them with a diagnosis. In addition, during the first data collection, researchers will be blind to the allocated grouping. As the study unfolds however, parent contact with the researchers may be required (e.g. reminders, assistance completing questionnaires). Consequently, blinding may be lost for some participants at post-test and follow up. The number and circumstance of participant unblinding will be recorded on an ongoing basis by the research manager as will any adverse outcomes or events from the research and/or Let’s Talk implementation. The control parents may also acquire some knowledge of the research aims, as the measures include parenting instruments. In addition, while control practitioners will not have received training in Let’s Talk, they will be aware of the aims of the research study and may inadvertently transmit this (verbally or through their behaviour) during treatment as usual to the parents in the control group.

Critically, the RCT design of the study occurs in an applied setting and involves three different sectors. Hence, the study design may be susceptible to fidelity and implementation problems. Given that a significant time lag exists in the translation of research to practice in mental health [19], the study will require a sound implementation framework across the various workforce sectors.

## Discussion

This program of research will evaluate the effectiveness of an existing evidence informed, brief family intervention (Let’s Talk About Children) delivered in adult mental health, community mental health and family service sectors. The research will address the question of whether the recovery journey of those living with a mental illness can be enhanced by acknowledging and addressing the parenting life domain. In particular, the study will systematically examine the evidence base for a recovery model that effectively addresses parenting, as measured by adult recovery (e.g. quality of life, hope and social inclusion), parenting competence and self-efficacy and family functioning. The parent recovery model may potentially offer additional mental illness prevention and/or early intervention benefits to children through minimising the risk factors associated with parental mental illness. Finally, the study will determine the economic value and cost-effectiveness of a larger roll out (longer term) of the parent recovery model for investment by Victorian and Australian governments.

## Abbreviations

ACTR: Australian and New Zealand Clinical Trials Register
GP: General Practitioner
RCT: Randomised Controlled Trial
SPSS: Statistical Package for Social Sciences
